# Three-dimensional cell culture can be regulated by vibration: low-frequency vibration increases the size of olfactory ensheathing cell spheroids

**DOI:** 10.1186/s13036-019-0176-1

**Published:** 2019-05-16

**Authors:** Lachlan J. Beckingham, Michael Todorovic, Johana Tello Velasquez, Marie-Laure Vial, Mo Chen, Jenny A. K. Ekberg, James A. St John

**Affiliations:** 1 The Clem Jones Centre for Neurobiology and Stem Cell Research, Brisbane, Australia; 2 Griffith Institute for Drug Discovery, Griffith University, Brisbane, Australia; 3 Menzies Health Institute Queensland, Griffith University, Gold Coast, Australia; 4 School of Nursing and Midwifery, Griffith University, Gold Coast, Australia

**Keywords:** Liquid marble, Glia, Migration, Transplantation, Amplitude

## Abstract

**Background:**

Olfactory ensheathing cell (OEC) transplantation is emerging as a promising therapy for spinal cord injuries. However, outcomes are inconsistent, and the method needs improvement. Currently, cells are injected into the injury site as a suspension, and often fail to form a three-dimensional (3D) network crucial for both survival of the transplanted cells, and for regeneration of severed axons. 3D culture systems are therefore likely to improve the method. Of the many 3D culture systems available, the spheroid-producing naked liquid marble (NLM) technique is particularly advantageous compared to other platforms as it rapidly generates cell spheroids which can easily be extracted for further handling. To improve production of the spheroids, we designed and tested a device which allows fine control over vibrational stimuli to liquid marble cell cultures. We applied vibrational frequencies of 20, 60, and 80 Hz with consistent amplitude to NLM containing OECs and assessed the size and number of the 3D cell spheroids generated as well as the migratory capacity of cells cultured in the vibrated spheroids.

**Results:**

Vibrating the NLMs led to fewer and dramatically larger spheroids in comparison to non-vibrated NLMs. Of the frequencies tested, 60 Hz caused over 70-fold increase in spheroid volume. When transferred to a culture plate, the larger spheroids retained their structure after 72 h in culture, and cells that migrated out of the spheroids covered a significantly larger area compared to cells migrating out of spheroids formed at all the other frequencies tested.

**Conclusions:**

We have shown that vibration can be used to regulate the formation of cell spheroids in NLM cultures. The ability to modulate the size of spheroids is useful for a range of 3D cell culture models and for preparing cells for in vivo transplantation.

## Background

Cell transplantation therapies show strong promise for treating neural injuries, even spinal cord injury (SCI) [1, 2]. Various cell types are being trialled for transplantation, in particular stem cells (embryonic, mesenchymal and neural) and glial cells such as Schwann cells and olfactory ensheathing cells (OECs) [3–8]. OECs in particular show great potential due to their ability to migrate long distances into scar tissue [9], promote axonal regeneration [10], interact with astrocytes [11], and phagocytose cell debris [12]. OEC transplantation has resulted in some remarkable regeneration in animals and humans with SCI [2, 8, 9, 13–15]. However, a major issue with these therapies is the low survival rate of the cells post-transplantation, likely due to the unfavourable microenvironment around the injury site [16], and the lack of support matrices in place to assist the cells [16, 17]. OECs require cell-cell contacts for many functions, in particular migration [18], and the lack of cell-cell contact in cell suspensions may be contributing to the low survival rate. Another problem that is hampering optimal development of OEC transplantation therapies is that many of the biological functions of these cells remain unknown. If we can further understand the normal biology of OECs, their behaviours can be further optimised towards better outcomes, in particular improved interactions between OECs and different cell types. Understanding the complex behaviours of OECs in a 3D environment is essential for this, and therefore a 3D culture system that mimics the in vivo milieu is highly warranted for research purposes.

Liquid marbles (LMs) are miniature bioreactors that are formed by coating droplets of cell seeded medium with a hydrophobic powder [19]. The lack of scaffolding commonly found in other 3D culturing systems allows for the self-assembly of cells into spheroids, which are small, spherical aggregates of proliferative and quiescent cells. Spheroid formation inside sessile LMs is highly variable, with a tendency for the cells to form non-uniform spheroid morphologies of various sizes, often with necrotic cores [20], and cell degradation at the edge of the aggregation [21]. This is due to the deformation of LMs when cultured on a hard surface. In a sessile marble, the cells will settle on the bottom, rendering this method relatively similar to a scaled down version of a low adhesion plate. Sessile marbles also have a tendency to evaporate [22], causing wrinkling which further hinders efficient spheroid production.

To combat this deformation and evaporation, we previously developed the floating LM method for OEC culture [21]. By floating the LMs on culture medium, the surface tension minimises marble deformation, and the medium also creates a humid microenvironment which prevents the majority of evaporation that occurs in sessile marbles. Additionally, a small air layer exists between the surface of the media and the LM that allows the LM to easily rotate due to natural vibrations. However, while the floating method has shown a vast improvement in the formation of uniform spheroid structures, there is still a large variability in size and number.

We have now developed a naked liquid marble (NLM) platform (Australian provisional patent 2,017,904,456, [23]) in which droplets of cell culture medium are incubated on a superhydrophobic coating. Inside the NLM platform, cells are free to interact with each other, forming multiple 3D spheroids that are uniform in size and shape in less than 24 h. To regulate the movement that occurs in NLMs, we have designed a device to apply a consistent and controllable source of sound-mediated vibration to the NLMs, and assessed how different frequencies affect the size and number of spheroids generated. This NLM system has been tested using not only OECs, but also human neural progenitor cells (ReNcell VM cell lines), as well as primary Schwann cells and Astrocytes [23]. This system has also shown to be comparable to other methods of producing spheroids.

After spheroids have been generated from NLMs, it is essential that the OECs are capable of migrating out of the spheroids to be able to interact appropriately with cells in the target tissue. It is, however, also advantageous if the original spheroid retains some of its structure in the long term, as a 3D network of cells is also present directly at the injury site. Thus, an ideal spheroid retains its structure over time, but cells are also capable of migrating out of the spheroid. We therefore assessed how vibration at different frequencies affected not only the number and size of spheroids, but also retention of spheroid structure and the ability of cells to migrate out of the spheroids.

## Results

### Calibration and amplitude distribution

**Fig. 1:**
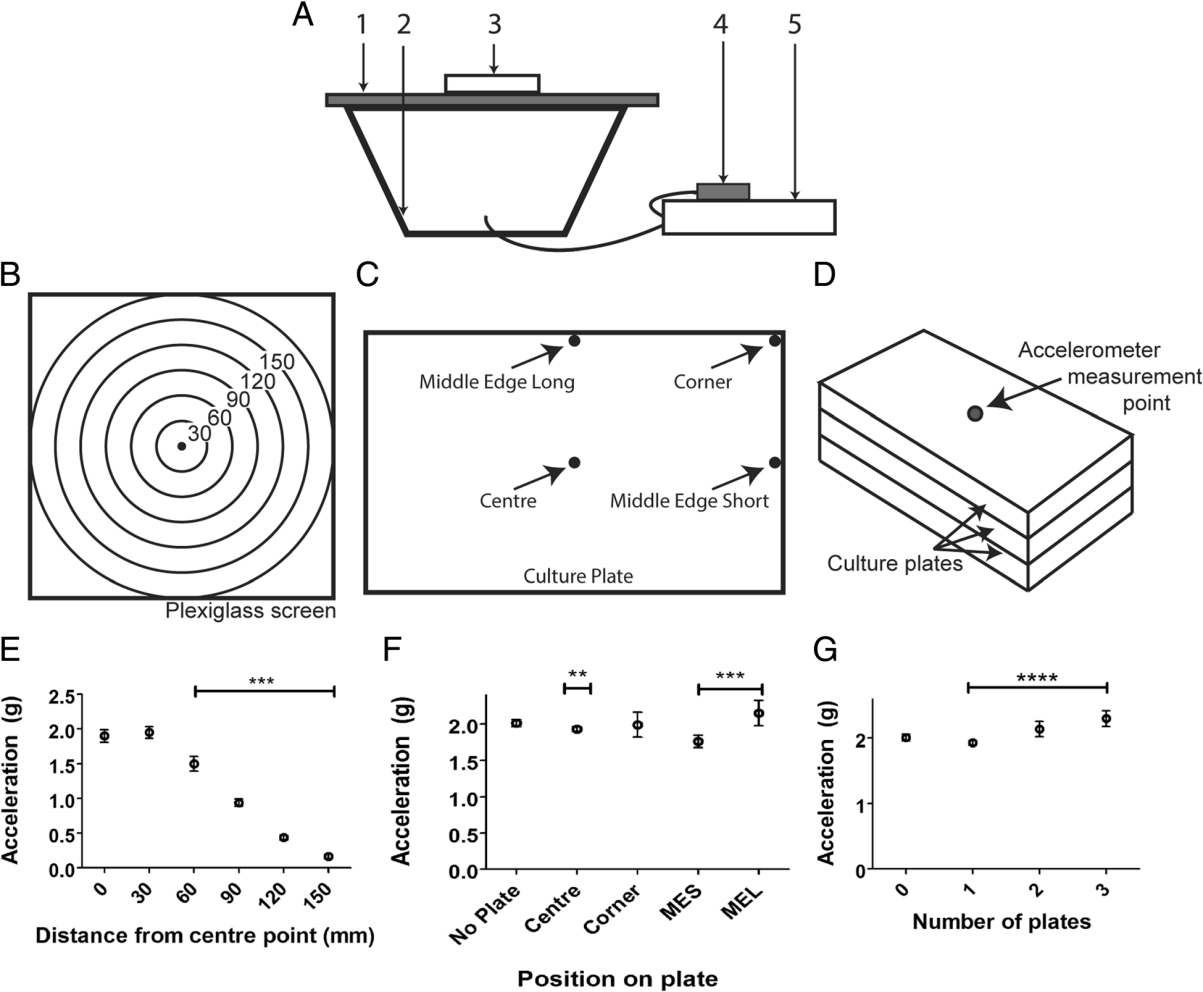
Acceleration distribution testing of the vibration apparatus. **a** The schematic shows the plexiglass screen [1] secured to the 12″ subwoofer cone [2], and a superhydrophobic culture plate [3] attached to the screen via adhesive tape. An MP3 playback device [4] produces the sinusoidal signal at the desired frequency, with a power amplifier [5] to drive the sinusoidal signal through the subwoofer. **b**-**d** Schematics showing positions of amplitude measurements on (**b**) the plexiglass screen (number indicate distance in mm from centre), (**c**) the cell culture plate and (**d**) plates stacked on top of each other. Graphs illustrate (**e**) distribution testing on the plexiglass screen of the rig (*** = *p* < 0.001), (**f**) plate distribution testing (** = *p* < 0.01 and *** = *p* < 0.001), and (**g**) plate stacking amplitude testing (**** = *p* < 0.0001). Data are mean ± SEM (One-Way ANOVA with Tukey’s Multiple Comparison test). MEL: *Middle Edge Long*; MES: *Middle Edge Short*

We hypothesised that vibration can be used to modify the properties of NLM cell cultures. To control the vibration frequency applied to NLM cultures of OECs, we designed a sound-driven vibration rig (Fig. 1a) suitable for subjecting the cultures to vibration at three frequencies (20, 60, and 80 Hz).

Calibration of the rig was performed by measuring the amplitude produced on the plexiglass screen. The amplitudes produced were within 6% of the calculated values, corresponding to an experimental amplitude of ±0.28 mm. The same values/ranges were recorded for all frequencies.

Testing of the amplitude across the screen (Fig. 1b) showed a radial distribution with a substantial decrease starting 60 mm out from the centre, culminating in 90% reduction of amplitude on the outer rim, 150 mm from the centre (Fig. 1e). Statistical analysis of the mean recorded values for all points on the plexiglass screen showed a significant difference at all points out from the centre (*p* < 0.01).

While there was variation across the screen, we next tested how this translated into culture plates placed on the screen. We measured the amplitude at different positions on the plates: in the centre for a base reading, in the corner, in the middle of the long edge of the rectangular plate (*Middle Edge Long*) and the middle of the short edge of the plate (*Middle Edge Short*) (Fig. 1c). All results were compared to the control reading with no plate at the centre of the screen. Plate variation testing revealed a 3.8% decrease in amplitude at the centre with one plate, a 12% decrease on the *Middle Edge Short*, and a 7% increase in amplitude on the *Middle Edge Long*. All of these readings were significantly different compared to the control reading with no plate (*p* < 0.01) (Fig. 1f). The amplitude at the corner of the plate was not significantly different compared to the control (*p* > 0.05).

Due to the variation in vibration across the screen which would render the use of multiple plates at the same time difficult, we tested whether stacking plates on top of one another would provide a method to allow multiple plates to be vibrated at the same time (Fig. 1d). An increase in amplitude totalling 11 and 19% was observed after the addition of the second and third plate, respectively, and the recorded values for the two additional plates were significantly different when compared to only one plate (*p* < 0.01) (Fig. 1g). These data show that plate stacking is not appropriate as it leads to inconsistent vibration amplitude amongst the plates. Therefore, for all following assays, a single cell culture plate was placed in the centre of the screen.

### Spheroid generation at test frequencies

**Fig. 2:**
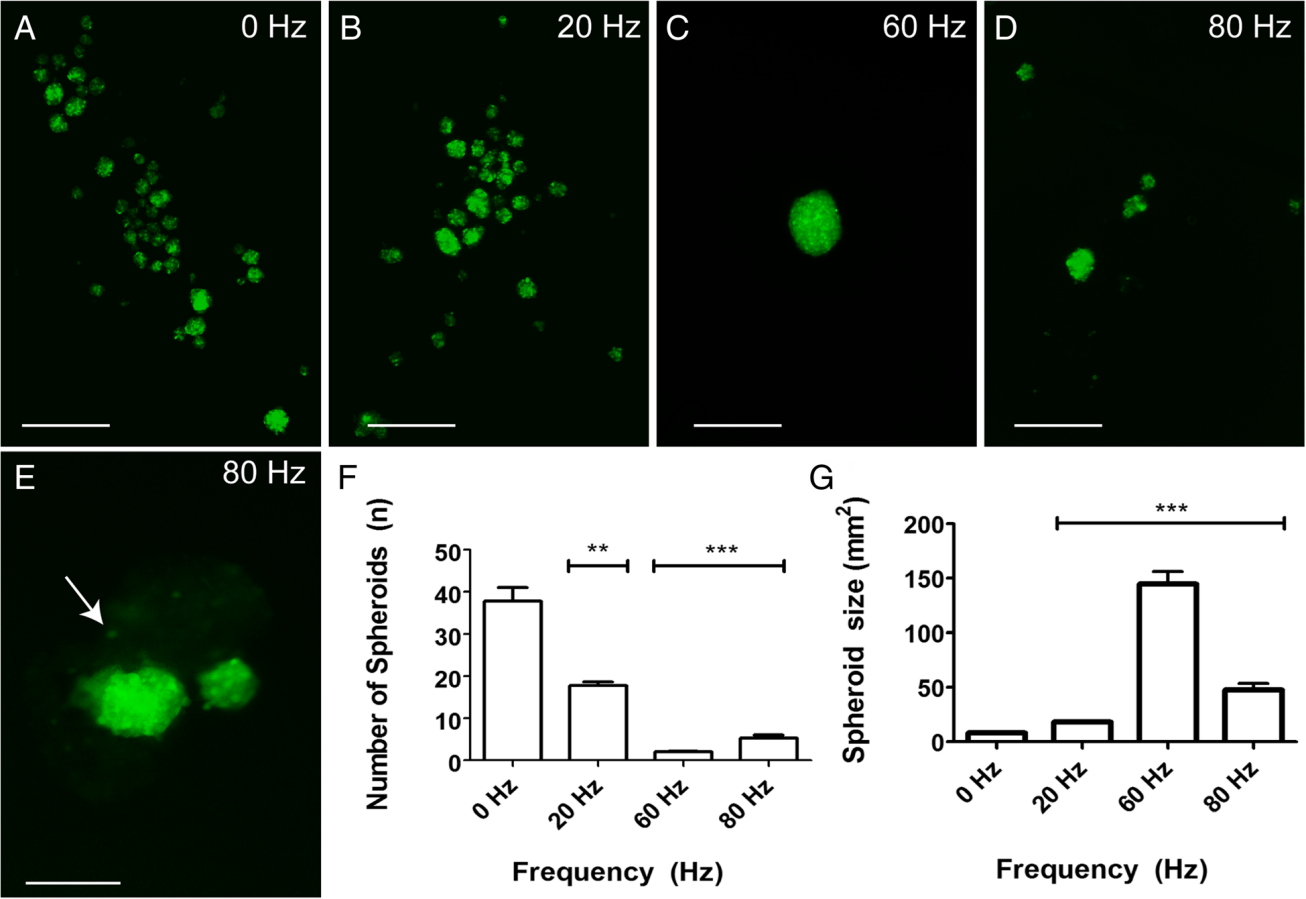
Vibration increases the size of spheroids generated from NLMs. **a**-**e**: Representative images of spheroids vibrated at test frequencies. **a** 0 Hz control, (**b**) 20 Hz, (**c**) 60 Hz, (**d**) 80 Hz, (**e**) 80 Hz frequency “fractured” spheroid with cell debris (arrow). **f**-**g** Bar graphs show (**f**) spheroid number and (**g**) spheroid cross-sectional area per NLM (*n* = 10 NLMs per test condition, 3 repeats). *** = *p* < 0.001 in both F and G compared to control, and each test condition compared to each other. Data presented are mean ± SEM (Kruskal-Wallis test and Dunn’s post-hoc test for multiple comparisons). Scale bars = 500 μm in **a**-**d**, 250 in **e**

To assess the effect of vibration on the formation of 3D spheroids, the device was used to induce vibration at different frequencies on NLMs (10 μL) seeded with 5000 OECs. In control non-vibrated NLMs, an average of 38 spheroids was produced per NLM, each with an average area of 8300 μm^2^ (Fig. 2a, f, g). Assuming that the spheroids were spherical, this area corresponds to a volume of ~ 0.0005 mm^3^ (V = 4/3·π·r^3^). When vibration was applied to the NLMs, there was a profound effect on the cross-sectional area and number of spheroids, with larger but fewer spheroids at all frequencies (Fig. 2a-d, f-g). The 20 Hz frequency produced an average of 18 spheroids with an average cross-sectional area of 18,000 μm^2^; 60 Hz produced an average of 2 spheroids with an average cross-sectional area of 145,000 μm^2^, and the 80 Hz frequency produced an average of 5 spheroids with an average cross-sectional area of 48,000 μm^2^ (*n* = 12 NLMs per test condition, 3 repeats). Significant differences were observed at all frequencies compared to the control for both number of spheroids produced (*p* < 0.001), and size (*p* < 0.001) (Fig. 2f, g). When comparing the different frequencies, there were significant differences in number and size between all different frequencies (*p* < 0.001 for all) (Fig. 2f, g). Again, if we assume the spheroids were spherical, these areas correspond to approximate volumes of 0.0018 mm^3^ (20 Hz), 0.0415 mm^3^ (60 Hz), and 0.0079 mm^3^ (80 Hz). The 60 Hz frequency was associated with the largest and fewest OEC spheroids produced by the NLMs, increasing spheroid volume 73-fold compared to control non-vibrated NLMs. At 80 Hz there were smaller spheroids compared to the 60 Hz, with fracturing occurring in some spheroids within the NLMs (arrow, Fig. 2e), but not all spheroids.

### Migration of cells out of spheroids

**Fig. 3:**
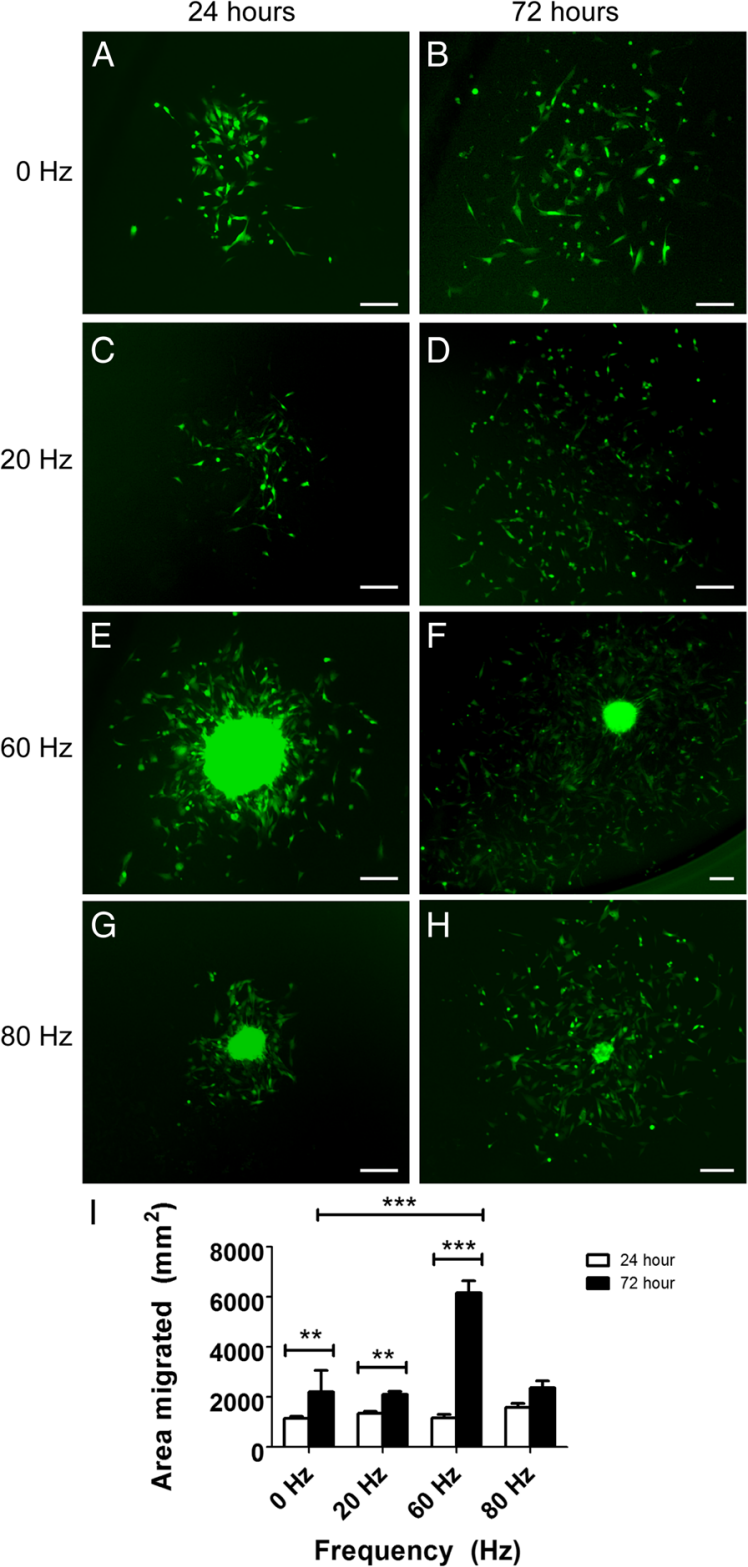
Effects of vibration on OEC migration. **a**-**f**): Representative images of spheroids 24 and 72 h after commencement of migration assay on a two-dimensional culture plate. **a** Control 24 h migration assay, spheroid completely dissociated. **b** Control 72 h migration assay, spheroid completely dissociated. **c** 20 Hz 24 h migration assay, spheroid completely dissociated. **d** 20 Hz 72 h migration assay, spheroid completely dissociated. **e** 60 Hz 24 h migration assay, spheroid not dissociated. **f** 60 Hz 72 h migration assay, spheroid partially dissociated. **g** 80 Hz 24 h migration assay, spheroid not dissociated. **h** 80 Hz 72 h migration assay, spheroid partially dissociated. **i** Bar graphs show the area occupied by migrating OECs at 24 h and 72 h for tested frequencies and control (no vibration), (*n* = 10 spheroids per condition, 3 repeats). Large horizontal bar indicates comparison between 60 Hz 72 h data and control 72 h data. Small horizontal bars indicate comparisons between 72 h and 24 h data for each test condition. ** = *p* < 0.01, *** = *p* < 0.001. Data presented is mean ± SEM (One-Way ANOVA with Tukey’s Multiple Comparisons test). Scale bars = 200 μm

The structure of the spheroids formed at 60 and 80 Hz suggested complex cell-cell interactions that may affect the ability of the spheroids to retain their structure over time and may also affect migration of cells out of the spheroids. To assess the structure stability and the migration of cells, we transferred the spheroids out of the NLMs into conventional cell culture plates to enable visualisation of the spheroids over time, and to allow the cells to attach and migrate onto the flask surface. After 24 h, control spheroids that had not been vibrated, and spheroids formed at the frequency of 20 Hz, dissociated completely and the cells had migrated out onto the surrounding well surface (Fig. 3a, c). In contrast, spheroids that had formed at frequencies of 60 and 80 Hz retained their structure and had not dissociated after 24 h (Fig. 3e, g). Under these conditions, cells had also migrated out onto the surrounding surface. No significant difference in the area occupied by migrating cells was observed among any of the test frequencies after 24 h (Fig. 3i). By 72 h the area occupied by migrating cells was significantly larger than at 24 h for all test groups, which was expected due to the additional time available for migration (Fig. 3i, small horizontal bars). However, cells from spheroids that had formed with 60 Hz vibration occupied a significantly larger area than migrating cells from all other groups (~ 2.6 times larger than control and 80 Hz, ~ 2.9 times larger than 20 Hz; *p* ≤ 0.001, Fig. 3i). The structure of the spheroids formed at 60 Hz was partially retained at 72 h (Fig. 3f), while the 80 Hz spheroids had almost completely dispersed (Fig. 3h).

Thus, vibration of NLMs at 60 Hz led to the generation of the largest spheroids; these spheroids retained their structure completely at 24 h and partially at 72 h after formation. Cells migrating out of these spheroids covered a larger surface area than cells migrating out of the spheroids generated at other frequencies at 72 h.

## Discussion

To date, only a few studies concerning the effects of vibration on cell culture have been reported. These mainly focussed on bone tissue differentiation using cultured osteoblasts [24], or sleep related studies on cell stress/inflammation markers using epithelial cells [25]. Studies have been performed on the effect of vibration on muscle [26] and bone tissue [27] for therapeutic purposes. There has, to our knowledge, only been one article published on the application of vibration in 3D cell culture [28]. In this study we used an apparatus similar to the novel setup used previously [25], and a novel 3D cell culture method (Australian Provisional Patent 2,017,904,456) [23] to assess the effect of low frequency vibration on 3D OEC spheroids. Our results demonstrate that vibration of the cell suspension cultures at 60 Hz led to the formation of large, compact cell spheroids. The fact that spheroids keep their integrity is crucial for the three-dimensional cell organisation, and thus significant for optimal cell transplantation. The larger spheroids, which contained more cells, then gave rise to more extensive migration of cells out of the spheroid. Thus the production of 3D cell spheroids under controlled vibration may be useful for preparing optimal cell constructs for transplantation.

**Fig. 4:**
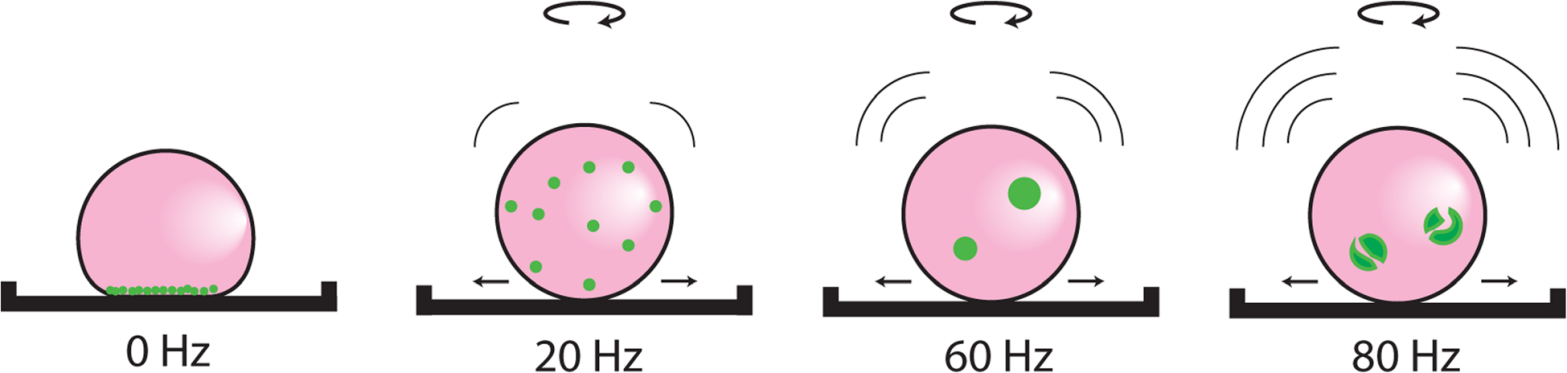
Schematic diagram of the effect of various vibrational frequencies on spheroid formation within the NLM system. Higher frequency vibration leads to larger, but fewer, spheroids, due to the constant motion of the NLM, which allows for more cell-cell interactions

The floating liquid marble method we had previously developed [21] was not suitable for vibrational application due to the fragility of the liquid marbles that were coated with the hydrophobic powder. Utilizing the newly developed NLM system, which can withstand considerable movement and shaking, we were able to successfully apply vibrational stimuli to liquid marble cell cultures. Our results suggest that the increased opportunity for cell-cell contact induced by the vibration leads to the formation of larger spheroids. It has been shown that OECs make stable cell-cell connections easily [18], so as they are given the opportunity to move around within the liquid marble via constant agitation, they gradually form larger spheroids as they encounter more and more cells (Fig. 4).

At the 60 Hz frequency, we observed over a 70-fold increase in spheroid size in comparison to the control, demonstrating that vibration has profound effects on spheroid size. We found that at 80 Hz (the highest frequency tested), the spheroids started to fracture and we concluded that 80 Hz is too violent a frequency for successful culturing of spheroids. We were unable to use a higher frequency (100 Hz) to further confirm this, as the equipment we were using was unable to achieve an amplitude of ±0.3 mm at 100 Hz. However, we can assume that 60 Hz is close to the upper limit tested of culturing with vibration without damage to the spheroids.

Vibration of NLMs containing OECs allows a successful way of modulating spheroid sizes, invaluable for in vitro studies of OEC behaviour. Being able to control spheroid size is also highly relevant for transplantation therapies, as the size of OEC spheroids can be tailored to individual types of injuries. An additional benefit may be an increased propensity for the cells of the spheroid to migrate in a cell-cell contact dependent manner [18]. As the spheroids are larger, with more extensive cell-cell contact, cell migration may be improved, which would greatly increase the therapeutic potential for SCI repair.

**Fig. 5:**
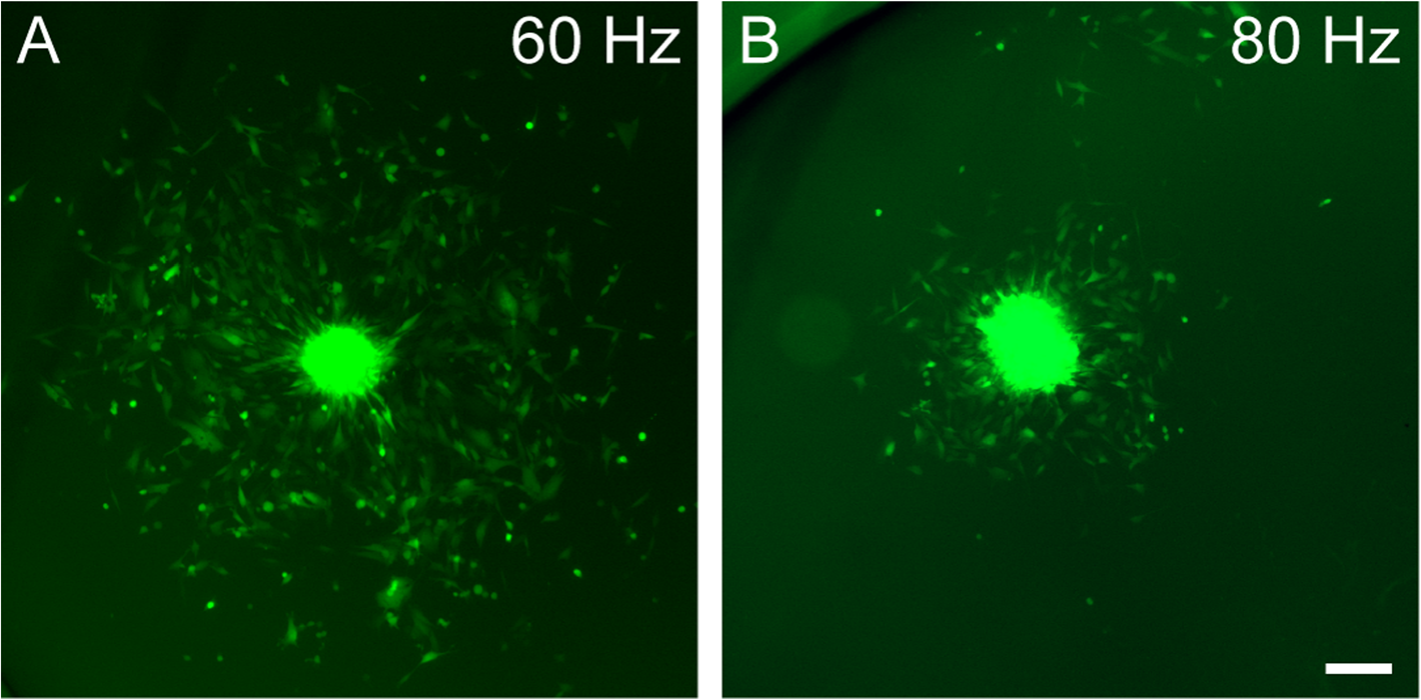
Comparison of the migration area after 72 h between two similar sized spheroids produced by **a**) 60 Hz, and **b**) 80 Hz. Scale bar = 200 μm

Our data showed that enhancing spheroid size significantly increased the area occupied by migrating cells around the spheroid. We theorize that this increase in migration is caused by structural differences within the spheroid, in that a 60 Hz spheroid may have a healthy core of constantly proliferating cells. This is evident when comparing two spheroids of the same size from different test conditions, (60 Hz versus 80 Hz), as we observe a significant increase in the migratory capacity of cells from the 60 Hz spheroid in comparison to the 80 Hz spheroid (Fig. 5). Due to the inherent size difference between the 60 Hz and 80 Hz spheroids, we were only able to find one pair of spheroids of even size to showcase this. This is relevant for therapeutic transplantation where migration of the transplanted cells into the host tissue is required. Another important aspect of generating larger spheroids is their longevity. Even after 72 h in culture, the largest spheroids, generated by 60 Hz vibrations, had still not dissociated completely. It is possible that cells may still be proliferating within the spheroid to keep the 3D structure intact over time. This may be ideal for transplantation purposes as cells within a 3D core structure of OECs is retained, providing a scaffold for regenerating axons, whilst large numbers of OECs also migrate into scar tissue. Whilst there is always concern that 3D cultures contain significant amounts of dead cells, we regard this as unlikely due to (1) the continual migration of cells out of the spheroid, showing that cells are alive and healthy, and (2) the fact that GFP, which is expressed by these cells, fluoresces poorly or not at all in dead cells. Thus, if there were significant amounts of dead cells in the spheroids, a large void zone with low fluorescence would be clearly visible.

## Conclusions

In conclusion, we have shown that careful application of low frequency vibration significantly affects the size and number of spheroids formed within NLMs. For certain frequencies, vibration also promotes the prolonged migratory rate of OECs out of spheroids. The potential benefits of this with regards to the use of spheroids as a treatment for SCI are (1) a platform for providing a range of spheroid sizes tailorable to any injury model, and (2) a demonstrated improved migration of OECs out of larger spheroids. Finally, controlling the size of 3D-cultured spheroids is highly advantageous for studying complex cell-cell interactions and cell behaviours in the laboratory. To successfully utilize this system clinically, large-scale production of spheroids would be required. This is feasible with the availability of commercial laboratory vibration/shaker plates, which could easily be applicable to this method.

## Methods

### Cell culture conditions

Previously generated immortalized mouse olfactory ensheathing cells (OECs) that express green fluorescent protein (GFP) were used [29]. Cells were maintained in complete medium containing 10% (vol/vol) fetal bovine serum (FBS) (Life Technologies), 10 μg mL^− 1^ basic fibroblast growth factor (FGF-2) (PeproTech), 10 ng mL^− 1^ epidermal growth factor (EGF) (PeproTech), 20 μg mL^− 1^ pituitary extract (Invitrogen), 2 μM forskolin (Sigma Aldrich), 2 mM gentamycin (Life Technologies), and Dulbecco’s Modified Eagle Medium: Nutrient Mix F12 (DMEM/F12) (Life Technologies). All cultures were incubated under standard incubation conditions of 37 °C and 5% CO_2_.

### Spheroid generation using naked liquid marbles (NLMs)

NLMs were generated according to our previously described method [23]. 500 cells/μL in 10 μL complete medium were aliquoted into superhydrophobic-coated, flat bottomed 96-well plates by hand to generate one NLM per well. Each plate could hold up to 96 marbles. For the purpose of these experiments, 12 × 10 μL marbles per plate were created for incubation under both vibration and control conditions (*n* = 12 NLMs per test condition, 3 replicates).

### Vibration rig

The apparatus (Fig. 1) used to generate sustained vibration was designed based on a previously published method [25]. Briefly, a sinusoidal signal driven by a power amplifier (Behringer) was played through a 12 in. subwoofer (Polkaudio). Culture plates were attached firmly with adhesive tape to a 3 mm thick plexiglass screen glued to the subwoofer cone, and the apparatus housed in an incubator under standard incubation conditions. NLMs were subjected to three vibrational frequencies (20, 60, and 80 Hz), and the apparatus was calibrated to an amplitude of ±0.3 mm using an accelerometer (Arduino) for each frequency tested. We were originally planning to test 100 Hz instead of 80 Hz, but found that we could not achieve the 100 Hz frequency without damaging out testing rig, thus the highest tested frequency was tuned back to 80 Hz. The equation used to determine optimal volume settings on the power amplifier for reaching the designated amplitude was:

$$ D=\frac{GA}{2{\pi}^2{F}^2} $$

where D = displacement (m), G = gravity (constant at 9.81 m/s^2^), A = acceleration (g), and F = frequency (Hz). A sample solution of the equation used to calibrate the rig to ±0.3 mm at 60 Hz is as follows:

$$ 0.0003=\frac{9.81\ast A}{2{\pi}^2{60}^2} $$

$$ A=2.173\ g $$

This amplitude was selected based on previous research in the field using a similar rig [25].

### Vibrational distribution testing

Prior to the generation of NLMs, the distribution of vibration across the plexiglass screen was tested using an accelerometer (Arduino), to determine whether there was any variation in amplitude at different points on the screen. The accelerometer was placed in the centre to provide a base reading, then at increments of 30 mm out until reaching the edge (150 mm). To determine the amplitude variation across a plate, the distribution of vibration across a plate centred on the screen was also tested. The accelerometer was placed in the centre for a base reading, and in the corner and middle of each edge of the plate, designated the terms *Middle Edge Long* and *Middle Edge Short*. All results were compared to the control reading with no plate at the centre of the screen. Finally, variation testing involving plate stacking was also performed. A baseline reading was taken from the centre of the screen, then a plate fixed to the rig with adhesive tape. A reading was taken, then another plate fixed. This process was repeated for up to three plates in total.

### Spheroid isolation and migration assays

After 24 h incubation, NLMs were transferred into a 10 mL Falcon tube, and 3 mL of complete medium was added to create a spheroid suspension. 10 μL of the spheroid suspension was transferred into each well of a 96-well plate and 50 μL of fresh complete medium was added. The plate was incubated for 24 h. A longer migration assay (72 h) was performed to evaluate the potential of larger spheroids generated via vibration to migrate continuously over a longer period of time.

### Image analysis

After a 24 h incubation period, spheroids were removed from the NLMs and transferred into a 96-well plate. Images were taken by an Olympus XM10 under an Olympus IX73 fluorescence microscope. ImageJ software was used to measure: (1) the cross-sectional area of the spheroids and (2) the area covered by the cells that had migrated out of the spheroids.

### Statistical analysis

Data normality was assessed and analysis were performed in SPSS using a Kruskal-Wallis Test with Dunn’s post hoc test or a one way ANOVA with Tukey’s Multiple Comparison post hoc test. All experiments were with triplicate independent assays (*n* = 3).

## Abbreviations

3D: Three-dimensional
LM: Liquid marble
NLM: Naked liquid marble
OEC: Olfactory ensheathing cell
SCI: Spinal cord injury
